# Supplementary material for: Modeling glioblastoma heterogeneity as a dynamic network of cell states
Source: Mol Syst Biol. 2021 Sep 16;17(9):e10105. doi: 10.15252/msb.202010105 (PMC8444284; doi:10.15252/msb.202010105)
Supplement: Supplementary file 5 — Source Data for Figure 3 [file MSB-17-e10105-s001.zip › Figure3A_sourcedata/GSEA_3065/hallmarks_state2.GseaPreranked.1623416269049/HALLMARK_UV_RESPONSE_DN.html]

Details for gene set HALLMARK\_UV\_RESPONSE\_DN[GSEA]

|  || Dataset | state2 |
| Phenotype | NoPhenotypeAvailable |
| Upregulated in class | na\_neg |
| GeneSet | HALLMARK\_UV\_RESPONSE\_DN |
| Enrichment Score (ES) | -0.59968483 |
| Normalized Enrichment Score (NES) | -2.3559453 |
| Nominal p-value | 0.0 |
| FDR q-value | 0.0 |
| FWER p-Value | 0.0 |
Table: GSEA Results Summary

  

Fig 1: Enrichment plot: HALLMARK\_UV\_RESPONSE\_DN      
 Profile of the Running ES Score & Positions of GeneSet Members on the Rank Ordered List

  

| PROBE | GENE SYMBOL | GENE\_TITLE | RANK IN GENE LIST | RANK METRIC SCORE | RUNNING ES | CORE ENRICHMENT || 1 | SRI |  |  | 181 | 0.293 | 0.0143 | No |
| 2 | APBB2 |  |  | 1130 | 0.110 | -0.0746 | No |
| 3 | DDAH1 |  |  | 1260 | 0.101 | -0.0768 | No |
| 4 | RBPMS |  |  | 1570 | 0.083 | -0.1004 | No |
| 5 | TJP1 |  |  | 1811 | 0.072 | -0.1178 | No |
| 6 | PRKAR2B |  |  | 1863 | 0.070 | -0.1152 | No |
| 7 | SFMBT1 |  |  | 2067 | 0.062 | -0.1298 | No |
| 8 | MTA1 |  |  | 2245 | 0.055 | -0.1424 | No |
| 9 | MGLL |  |  | 2293 | 0.054 | -0.1412 | No |
| 10 | ADD3 |  |  | 2401 | 0.051 | -0.1469 | No |
| 11 | MAP2K5 |  |  | 2630 | 0.043 | -0.1663 | No |
| 12 | YTHDC1 |  |  | 2727 | 0.041 | -0.1719 | No |
| 13 | SLC7A1 |  |  | 3014 | 0.033 | -0.1987 | No |
| 14 | MIOS |  |  | 3308 | 0.027 | -0.2269 | No |
| 15 | NR1D2 |  |  | 3471 | 0.024 | -0.2414 | No |
| 16 | AGGF1 |  |  | 3740 | 0.020 | -0.2679 | No |
| 17 | MYC |  |  | 3842 | 0.018 | -0.2766 | No |
| 18 | CAP2 |  |  | 3862 | 0.018 | -0.2766 | No |
| 19 | CDKN1B |  |  | 4019 | 0.015 | -0.2915 | No |
| 20 | MRPS31 |  |  | 4123 | 0.014 | -0.3010 | No |
| 21 | DYRK1A |  |  | 4284 | 0.011 | -0.3169 | No |
| 22 | RASA2 |  |  | 4350 | 0.010 | -0.3228 | No |
| 23 | ICA1 |  |  | 4373 | 0.009 | -0.3241 | No |
| 24 | NFIB |  |  | 4378 | 0.009 | -0.3234 | No |
| 25 | PIAS3 |  |  | 4491 | 0.007 | -0.3346 | No |
| 26 | HAS2 |  |  | 4545 | 0.007 | -0.3395 | No |
| 27 | TENT4A |  |  | 4548 | 0.007 | -0.3390 | No |
| 28 | SMAD3 |  |  | 4579 | 0.006 | -0.3415 | No |
| 29 | RXRA |  |  | 4592 | 0.006 | -0.3421 | No |
| 30 | DUSP1 |  |  | 4653 | 0.005 | -0.3480 | No |
| 31 | VAV2 |  |  | 4836 | 0.002 | -0.3672 | No |
| 32 | AMPH |  |  | 4872 | 0.001 | -0.3708 | No |
| 33 | ZMIZ1 |  |  | 5050 | -0.001 | -0.3897 | No |
| 34 | SPOP |  |  | 5080 | -0.001 | -0.3926 | No |
| 35 | DMAC2L |  |  | 5137 | -0.002 | -0.3984 | No |
| 36 | BCKDHB |  |  | 5263 | -0.004 | -0.4113 | No |
| 37 | NFKB1 |  |  | 5273 | -0.004 | -0.4117 | No |
| 38 | NIPBL |  |  | 5376 | -0.006 | -0.4219 | No |
| 39 | PHF3 |  |  | 5455 | -0.008 | -0.4294 | No |
| 40 | AKT3 |  |  | 5783 | -0.013 | -0.4629 | No |
| 41 | INSIG1 |  |  | 5815 | -0.013 | -0.4647 | No |
| 42 | FYN |  |  | 6106 | -0.018 | -0.4937 | No |
| 43 | SCHIP1 |  |  | 6274 | -0.021 | -0.5091 | No |
| 44 | BMPR1A |  |  | 6281 | -0.021 | -0.5073 | No |
| 45 | MAPK14 |  |  | 6426 | -0.024 | -0.5200 | No |
| 46 | ADORA2B |  |  | 6618 | -0.028 | -0.5373 | No |
| 47 | NR3C1 |  |  | 6802 | -0.032 | -0.5532 | No |
| 48 | RND3 |  |  | 6928 | -0.035 | -0.5626 | No |
| 49 | SIPA1L1 |  |  | 7046 | -0.038 | -0.5708 | No |
| 50 | SCAF8 |  |  | 7158 | -0.041 | -0.5780 | No |
| 51 | CDC42BPA |  |  | 7216 | -0.042 | -0.5792 | No |
| 52 | PRDM2 |  |  | 7236 | -0.043 | -0.5763 | No |
| 53 | ATRX |  |  | 7408 | -0.047 | -0.5892 | No |
| 54 | RUNX1 |  |  | 7507 | -0.051 | -0.5939 | Yes |
| 55 | ID1 |  |  | 7519 | -0.051 | -0.5892 | Yes |
| 56 | CELF2 |  |  | 7549 | -0.052 | -0.5863 | Yes |
| 57 | ARHGEF9 |  |  | 7560 | -0.052 | -0.5814 | Yes |
| 58 | F3 |  |  | 7662 | -0.055 | -0.5859 | Yes |
| 59 | PDLIM5 |  |  | 7663 | -0.055 | -0.5796 | Yes |
| 60 | FZD2 |  |  | 7754 | -0.058 | -0.5826 | Yes |
| 61 | ACVR2A |  |  | 7795 | -0.059 | -0.5800 | Yes |
| 62 | MAGI2 |  |  | 7798 | -0.060 | -0.5734 | Yes |
| 63 | ERBB2 |  |  | 7902 | -0.063 | -0.5772 | Yes |
| 64 | PLPP3 |  |  | 7911 | -0.063 | -0.5708 | Yes |
| 65 | BDNF |  |  | 8073 | -0.070 | -0.5800 | Yes |
| 66 | VLDLR |  |  | 8186 | -0.077 | -0.5832 | Yes |
| 67 | ANXA4 |  |  | 8188 | -0.077 | -0.5745 | Yes |
| 68 | PTEN |  |  | 8202 | -0.077 | -0.5670 | Yes |
| 69 | EFEMP1 |  |  | 8240 | -0.079 | -0.5618 | Yes |
| 70 | SLC22A18 |  |  | 8276 | -0.081 | -0.5562 | Yes |
| 71 | CDK13 |  |  | 8289 | -0.082 | -0.5481 | Yes |
| 72 | CITED2 |  |  | 8322 | -0.084 | -0.5419 | Yes |
| 73 | NOTCH2 |  |  | 8358 | -0.086 | -0.5357 | Yes |
| 74 | KALRN |  |  | 8547 | -0.100 | -0.5444 | Yes |
| 75 | SYNE1 |  |  | 8557 | -0.100 | -0.5339 | Yes |
| 76 | ATP2B1 |  |  | 8584 | -0.102 | -0.5249 | Yes |
| 77 | COL11A1 |  |  | 8598 | -0.103 | -0.5145 | Yes |
| 78 | KCNMA1 |  |  | 8649 | -0.107 | -0.5075 | Yes |
| 79 | GJA1 |  |  | 8728 | -0.114 | -0.5028 | Yes |
| 80 | MET |  |  | 8800 | -0.122 | -0.4963 | Yes |
| 81 | BHLHE40 |  |  | 8804 | -0.123 | -0.4826 | Yes |
| 82 | PIK3R3 |  |  | 8840 | -0.127 | -0.4717 | Yes |
| 83 | DLG1 |  |  | 8857 | -0.130 | -0.4584 | Yes |
| 84 | ATP2C1 |  |  | 8879 | -0.133 | -0.4454 | Yes |
| 85 | ANXA2 |  |  | 8891 | -0.134 | -0.4312 | Yes |
| 86 | MMP16 |  |  | 8897 | -0.135 | -0.4162 | Yes |
| 87 | ATRN |  |  | 8912 | -0.137 | -0.4019 | Yes |
| 88 | TGFBR2 |  |  | 8916 | -0.137 | -0.3865 | Yes |
| 89 | MAP1B |  |  | 8945 | -0.141 | -0.3733 | Yes |
| 90 | ATXN1 |  |  | 8996 | -0.148 | -0.3616 | Yes |
| 91 | SDC2 |  |  | 9002 | -0.149 | -0.3450 | Yes |
| 92 | TFPI |  |  | 9060 | -0.159 | -0.3328 | Yes |
| 93 | PRKCA |  |  | 9072 | -0.161 | -0.3154 | Yes |
| 94 | LDLR |  |  | 9117 | -0.170 | -0.3006 | Yes |
| 95 | CAV1 |  |  | 9130 | -0.172 | -0.2821 | Yes |
| 96 | IGF1R |  |  | 9152 | -0.178 | -0.2639 | Yes |
| 97 | LAMC1 |  |  | 9286 | -0.226 | -0.2522 | Yes |
| 98 | ATP2B4 |  |  | 9314 | -0.243 | -0.2271 | Yes |
| 99 | COL5A2 |  |  | 9316 | -0.244 | -0.1992 | Yes |
| 100 | SYNJ2 |  |  | 9337 | -0.259 | -0.1716 | Yes |
| 101 | PMP22 |  |  | 9347 | -0.271 | -0.1414 | Yes |
| 102 | CACNA1A |  |  | 9428 | -0.519 | -0.0904 | Yes |
| 103 | IGFBP5 |  |  | 9438 | -0.797 | 0.0002 | Yes |
Table: GSEA details [plain text format]

  

Fig 2: HALLMARK\_UV\_RESPONSE\_DN: Random ES distribution      
 Gene set null distribution of ES for **HALLMARK\_UV\_RESPONSE\_DN**
